# Current approaches using remote monitoring technology in alcohol use disorder (AUD): an integrative review

**DOI:** 10.1093/alcalc/agaf032

**Published:** 2025-06-12

**Authors:** Valentina Navarro-Ovando, Sterre van Schie, Imme Garrelfs, Jop Rijksbaron, Cristian Rodriguez Rivero, Ron Mathôt, Glenn Dumont

**Affiliations:** Amsterdam UMC Location University of Amsterdam, Department of Hospital Pharmacy and Clinical Pharmacology, Meibergdreef 9, 1105 AZ, Amsterdam, The Netherlands; Amsterdam Public Health, Amsterdam, The Netherlands; ADHDcentraal, Janskerkhof 16, 3512 BM, Utrecht, The Netherlands; Amsterdam UMC, location University of Amsterdam, Meibergdreef 9, 1105 AZ, Amsterdam, The Netherlands; ADHDcentraal, Janskerkhof 16, 3512 BM, Utrecht, The Netherlands; Centre for Engineering Research in Intelligent Sensors and Systems (CeRISS), Cardiff Metropolitan University Cardiff, Western Avenue CF5 2YB, Cardiff, Wales, United Kingdom; Universitat Politècnica de Catalunya Barcelona Tech (UPC), Department of Mining, Industrial and ICT Systems Engineering, Carrer de Jordi Girona 31, Les Corts, 08034, Barcelona, Spain; Amsterdam Public Health, Amsterdam, The Netherlands; Amsterdam UMC Location University of Amsterdam, Department of Hospital Pharmacy and Clinical Pharmacology, Meibergdreef 9, 1105 AZ, Amsterdam, The Netherlands; Amsterdam Public Health, Amsterdam, The Netherlands; ADHDcentraal, Janskerkhof 16, 3512 BM, Utrecht, The Netherlands

**Keywords:** alcohol use disorder, AUD, mHealth, remote monitoring, digital therapeutics, near-continuous monitoring

## Abstract

**Aims:**

This integrative review aims to synthesize and update the current literature on mHealth applications and devices, such as smartphones, wearables, and breathalyzers, in alcohol use disorder (AUD) monitoring. It discusses the evolution of these tools, the current level of evidence, and facilitators and barriers to their implementation in research and interventions. The future potential for personalized interventions is also explored.

**Methods:**

Integrative review. Three databases—PubMed, Web of Science, and PsycINFO—were used to identify quantitative and qualitative English written publications between 2014 and 2024, using terms related to mHealth, remote monitoring, and AUD. Results were extracted and comprehensively presented by topic.

**Results:**

Fifty-eight studies were included in the synthesis. Smartphones, mobile phones, breathalyzers, and wearables with transdermal sensors are the most frequently used devices for remote monitoring. The included studies demonstrated varying levels of development and evidence across devices. Ecological Momentary Assessment via smartphones was the most frequently used and, along with breathalyzers, was successfully applied in clinical trials involving interventions. Wearables were scarcely tested in interventions. Challenges related to adherence and psychological factors remain in a longer period of monitoring. Incipient use of predictive models integrating ongoing data shows promise in informing care providers and optimizing intervention delivery.

**Conclusions:**

Evidence supporting mHealth tools for AUD remains uneven across device types. While smartphones and breathalyzers show greater clinical applicability, wearables and passive sensing remain exploratory. Robust, comparative research is needed to guide effective selection and integration into care.

## Teaser

**Short Summary:** mHealth tools enhance remote monitoring in AUD interventions, though development varies. Smartphones are the most frequently reported devices, followed by breathalyzers and, lastly, wearables. Extended use of smartphone apps and breathalyzers has been documented. Challenges include missing data and sustained adherence. Predictive models show promise in optimizing information use from real-time and near-real-time data.

## Introduction

Alcohol use disorder (AUD) poses a significant global health matter, with a prevalence of 14.8% among males and 3.5% among females in Europe (WHO 2018a). Treatment for AUD faces persisting challenges, including dropout rates of 19%–34% and remission failure in 10%–18% of cases (Lappan *et al*. 2020, Maisto *et al*. 2020). Even among those achieving remission, relapse remains a concern. Approximately 60% of patients aiming for abstinence experienced a drinking episode within 6 months of treatment (Sliedrecht *et al*. 2019). Furthermore, 25% of individuals aiming to reduce their consumption continued to engage in moderate- to high-risk drinking (Tuithof *et al*. 2013).

Although most AUD cases are transient (Tuithof *et al*. 2013, Seeley *et al*. 2019), fewer than 25% of individuals with persistent AUD seek treatment, often delaying care until the disorder is well established or other alcohol-related conditions emerge (Tuithof *et al*. 2016, Carvalho *et al*. 2019). Interventions that reduce alcohol consumption significantly improve health outcomes. A reduction by just one World Health Organization (WHO) risk category leads to significant decrease in mortality and chronic disease risk (WHO 2000, Witkiewitz *et al*. 2016). Furthermore, a sustained reduction of two or more levels can reduce healthcare costs by up to 52% in the first year and 44% over 3 years (Aldridge *et al*. 2021).

The implementation of emerging mobile health (mHealth) technologies in AUD treatment shows promise in facilitating sustained reductions in alcohol consumption during and after treatment. Unlike traditional computer-based interventions, mHealth enables remote monitoring through wireless devices, providing near real-time updates on a patient’s status (WHO 2018b, Marsch *et al*. 2020). These constant updates allow for the monitoring of highly dynamic factors that contribute to lapses and relapses (Morgenstern *et al*. 2014). The lapse/relapse process often begins with a vulnerable state, followed by high-risk situations that trigger drinking (Witkiewitz and Marlatt 2007). Identifying high-risk circumstances enables the potential for timely support to prevent adverse outcomes (Marsch *et al*. 2014). Additionally, mHealth technologies can help overcome healthcare operational limitations by maintaining treatment gains and reducing the need for more intensive interventions due to relapse (Hutschemaekers *et al*. 2007, McKay 2021).

Despite extensive research evidence of digital therapeutics in addiction, there has been limited focus on the integration of mHealth into AUD treatment, specifically. A 2022 meta-review identified 555 studies on substance use disorders (SUDs) where only 60 involved mHealth (Philippe *et al*. 2022). A recent review of 3056 SUD studies listed 251 studies incorporating mHealth for monitoring or intervention in AUD (Johansson *et al*. 2024). However, no reviews have comprehensively updated on the different mHealth approaches for the advancements in remote monitoring for AUD treatment, including different levels of evidence.

This integrative review aims to synthesize current literature on mobile technology–based remote monitoring systems for AUD, exploring the integration of mobile device data into therapeutic processes. It covers the evolution of mHealth applications in alcohol addiction, their applications in monitoring and intervention, and their potential for future tailored interventions. We expect that providing an update overview on the broad development of mHealth in AUD will contribute to informing researchers and clinicians about the state of the art of this rapidly evolving topic.

## Materials and methods

This study followed an integrative review approach to synthesize existing literature on mHealth technologies for near-continuous remote monitoring in AUD treatment. Integrative reviews incorporate both quantitative and qualitative studies, providing a comprehensive understanding of a research topic.

### Search strategy

Three databases, PubMed, Web of Science, and PsycINFO, were searched using terms related to digital health/mHealth, remote monitoring, and AUD in titles and abstracts. The search was conducted on 11 June 2024, with filters applied to include studies published between 2014 and 2024, in English, involving human adults (19+ years). Reference lists were also manually screened. Details of the search terms are listed in Table 1.

**Table 1:** List of terms used per concept. The table displays the key concepts and corresponding search terms used for the literature search

**Concepts**	**Search terms**
*Digital health/mHealth*	Digital health, mHealth, mobile health, mobile technology, smartphone, mobile phone, cell phone, mobile applications, digital biomarkers
*Remote monitoring*	Monitoring, remote monitoring, wearable, smart watch, breathalyzer
*Alcohol use disorder*	Alcohol dependence, alcohol abuse, alcoholism, alcohol-related disorders, binge drinking.

Included studies focused on the implementation of digital health tools for remote alcohol consumption monitoring or related outcomes in real-world settings, meaning naturalistic, everyday environments outside controlled research or clinical contexts. Eligible populations included individuals with a formal diagnosis of AUD or those who scored above a clinically meaningful threshold on validated screening tools, indicating a high risk of presenting AUD. Studies included other stakeholders in AUD care (e.g. clinicians) were also included, as well as qualitative studies focused on the perception toward the use of mHealth in AUD interventions.

Exclusion criteria comprised studies where alcohol use was secondary to another SUD (i.e. alcohol consumption was better accounted for by the use or withdrawal of another substance), lacked standardized measures of AUD symptoms, or did not involve near-continuous monitoring. Near-continuous monitoring was defined as involving repeated daily measures or high-frequency sampling through methods like prompt questions, smartphone sensors, and direct alcohol measures via mobile or wearable biosensors. This criterion was intended to identify studies capable of capturing the dynamics of AUD factors over time.

**Figure 1:**
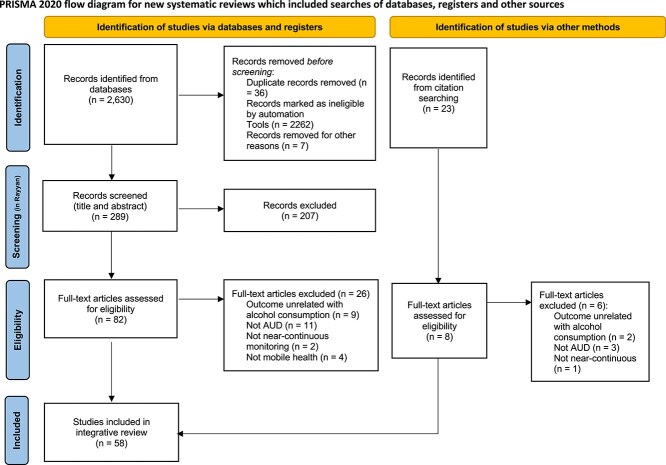
Prisma flow diagram of the study selection process. The chart displays the selection process from databases and other sources. Adapted from Page *et al.* (2021).

Figure 1 depicts the study selection process. Results were exported to the Rayyan platform for the title and abstract screening. Two authors participated in the full-text screening and selection (V.N., I.G.), and one participated in the validation (S.V.S.). Discrepancies were discussed until consensus was reached. Information was extracted into a Microsoft Excel spreadsheet.

## Results

Table 2 summarizes key characteristics of the included studies. Among the 58 included studies, 43% focused on using monitoring to collect information, while 57% included additional therapeutic components or clinical decision-making based on monitoring. Key topics included usability, feasibility, acceptability, effectiveness, engagement, and adherence, with fewer studies examining predictors of alcohol consumption.

### General results overview

#### Devices

In terms of the type of device utilized, most studies involved smartphones/mobile phones (*N* = 44) and 27 only used these devices, relying on pre-installed smartphone applications or text message exchange. Breathalyzers (*N* = 19) were included more frequently compared with wearables (*N* = 11). The deployment of both breathalyzers and wearables was primarily utilized as a monitoring instrument for alcohol consumption, by measuring breath alcohol concentration (BrAC) and transdermal alcohol concentration (TAC). Two studies used wearables for the monitoring of additional variables, including stress and physical activity. One study used a smartphone with a pill bottle equipped with a chip-enabled cap to record instances of opening.

#### Population

Most studies (31) included participants with a confirmed diagnosis of AUD. Few studies included specific groups such as adults with human immunodeficiency virus (HIV), those with alcohol-related liver disease, experiencing homelessness, and only females. Additionally, two studies involved clinicians working in AUD care.

#### Monitoring period and main outcomes

**Figure 2:**
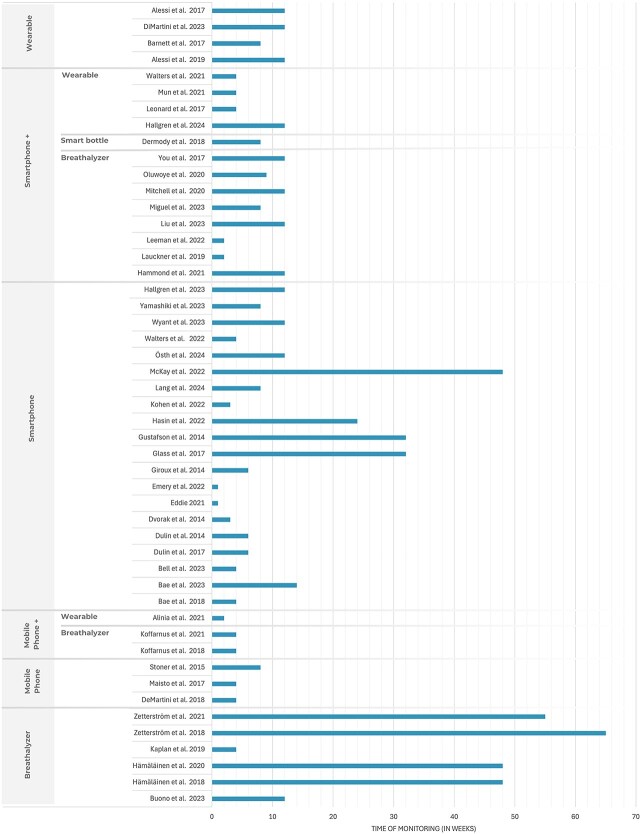
Monitoring period per study and corresponding devices used. The timeline illustrates the duration of monitoring for each study, categorized by the type of device used for data collection.

Given primary constraints associated with mHealth pertain to patient engagement over extended periods of intervention, we have extracted the monitoring duration for each study. Monitoring durations ranged from 1 to 65 weeks, with 12 weeks being the most common (Fig. 2). Longer monitoring phases were primarily retrospective studies based on clinical data files or randomized controlled trials (RCTs) focused on continuing care and relapse prevention.

**Table 2:** Summary of included articles organized by device. The table summarizes research articles, providing details about the type of study design, population size, monitoring tools or interventions used, the source of alcohol monitoring, and the main outcome measure

**Study**	**Type of research**	**Population** ***N*(% female)**	**Types of monitoring tools or interventions**	**Source of alcohol monitoring** ^**a**^	**Main outcome measure**
(Hämäläinen *et al*. 2018)	Single-arm predictive design	*N* = 30(36.7%) AUD diagnosis, outpatient treatment, and aftercare postin-patient treatment	Breathalyzer, web-based intervention	BrAC and blood (PEth)	Alcohol consumption
(Hämäläinen *et al*. 2020)	Randomized controlled trial	*N* = 115(34%) AUD diagnosis, outpatient treatment, and aftercare postin-patient treatment	Breathalyzer, web-based intervention	BrAC and blood (PEth)	Alcohol consumption
(Wallden *et al*. 2024)	Retrospective study	*N* = 1809(?%) AUD diagnosis, data collected from real-world clinical practice	eHealth system with a breathalyzer, an app for patients, and a caregiver portal	BrAC	Alcohol consumption
(Zetterström *et al*. 2018)	Retrospective study	*N* = 54(37%) AUD diagnosis, outpatient treatment, and aftercare postin-patient treatment	Breathalyzer	BrAC, blood (PEth), self-report (TLFB)	Alcohol consumption
(Kaplan and Koffarnus 2019)	Randomized parallel trial (two-armed)	*N* = 39(31%) AUD diagnosis, treatment-seeking	Daily reports (day before) breathalyzer	BrAC	Alcohol measures
(Gordon *et al*. 2017)	Qualitative	*N* = 9(?%) Clinicians and clinical researchers with extensive experience in AUD treatment	Breathalyzer	BrAC	Clinical guidelines
(Buono *et al*. 2023)	Randomized controlled trial	*N* = 96(18.75%) AUD diagnosis, outpatient treatment	Breathalyzer	BrAC	Motivation
(Zetterström *et al*. 2021)	Retrospective study	*N* = 1768(?%) AUD diagnosis, data from previous clinical trials and real-world clinical practice	Digital biomarker	BrAC, blood (PEth)	Validation of other measures
(Maisto *et al*. 2017)	Prospective observational study	*N* = 119(42%) AUD diagnosis, outpatient treatment	EMA (4/day)	Self-report (daily)	Alcohol consumption
(Stoner *et al*. 2015)	Randomized controlled trial	*N* = 79(34,2%) AUD diagnosis, treatment seeking	Smartphone app, reminders text messages	Self-report (daily)	Medication adherence
(DeMartini *et al*. 2018)	Randomized controlled trial	*N* = 15(27%) Liver transplant candidates with alcohol-related liver disease	Text messages, EMA (3/week)	Self-report (TLFB), BrAC, urine (EtG)	User experience and adherence
(Koffarnus *et al*. 2018)	Randomized parallel trial (two-armed)	*N* = 40(30%) AUD diagnosis, treatment seeking	Contingency management intervention using a breathalyzer	BrAC	Alcohol consumption
(Koffarnus *et al*. 2021)	Randomized parallel trial (two-armed)	*N* = 36(58%) AUD diagnosis, treatment-seeking	Contingency management intervention using a breathalyzer	BrAC	Alcohol consumption
(Wright *et al*. 2018)	Randomized controlled trial	*N* = 269(51%) Risky drinking behavior (≥7/10 units in a single occasion), nontreatment-seeking young adults	HR-EMA (hourly/planned drinking day) questionnaires, text messaging	Self-report (1 month recall)	Alcohol consumption
(Alinia *et al*. 2021)	Proof-of-concept study	*N* = 11(60%) AUD, in early recovery posthospitalization/acute care	Wristband for stress monitoring and smartphone app	Self-report (every 2 days)	Validation of other measures
(Bae *et al*. 2018)	Prospective observational study	*N* = 38(39%) AUDIT-C score ≥3/4+ at least one binge drinking occasion (>4/5 units), nontreatment seeking	Smartphone app sensors, EMA (2/week)	Self-report (daily)	Alcohol consumption
(Bae *et al*. 2023)	Single-arm predictive design	*N* = 75(71%) AUDIT-C score ≥3/4+ at least one binge drinking occasion (>4/5 units), nontreatment seeking	Smartphone app sensors	Self-report (daily)	Alcohol consumption
(Dulin *et al*. 2014)	Pilot single-arm study	*N* = 28(48%) Meeting AUD criteria, interested in changing their drinking patterns	Smartphone app	Self-report (daily)	Alcohol consumption
(Dulin *et al*. 2017)	Nonrandomized parallel trial	*N* = 28(48%) Meeting AUD criteria, interested in changing their drinking patterns	Smartphone app	Self-report (daily)	Alcohol consumption
(Eddie *et al*. 2021)	Prospective observational study	*N* = 42(38.1%) Meeting AUD criteria, outpatient treatment	EMA (3/day × 3 h)	Self-report (TLFB)	Alcohol consumption
(Gustafson *et al*. 2014)	Randomized controlled trial	*N* = 349(39%) Meeting alcohol dependence criteria (DSM-IV), continuing care	Smartphone app	Self-report (1 month recall)	Alcohol consumption
(Hasin *et al*. 2022)	Randomized parallel trial (three-armed)	*N* = 114(42%) People living with HIV and meeting alcohol dependence criteria (DSM-IV), HIV clinic patients	Daily report of consumption, motivational interventions	Self-report (daily)	Alcohol consumption
(Kohen *et al*. 2022)	Prospective observational study	*N* = 155(59%) Moderate-high risk consumption (AUDIT-C mean score = 5.9), half met AUD criteria, nontreatment seeking	EMA (~4/day)	Self-report (daily)	Alcohol consumption
(McKay *et al*. 2022)	Randomized controlled trial	*N* = 262(29.29%) Meeting AUD criteria, continuing care after intensive outpatient program	Continuing care. phone calls, smartphone app	Self-report (daily), blood (%dCDT)	Alcohol consumption
(Yamashiki *et al*. 2023)	Pilot single-arm study	*N* = 14(43%) Alcohol-related liver disease, outpatient care	Smartphone app	Self-reported (daily), blood (GGT, GGT-CDT)	Alcohol consumption
(Walters *et al*. 2022)	Pilot single-arm trial	*N* = 41(19.5%) AUDIT score ≥8, receiving health services at a homeless shelter	JITAI	Self-report (daily)	Alcohol consumption
(Emery *et al*. 2022)	Prospective observational study	*N* = 42(38.1%) Meeting AUD criteria, outpatient treatment	EMA (random and self-initiated)	Self-report (TLFB)	Discrete emotions
(Dvorak *et al*. 2014)	Prospective observational study	*N* = 74(58.11%) Moderate–high problematic drinking (AUDIT score = 5–27), young adults, nontreatment seekers	Smartphone app^a^, in a media player	Self-report (daily)	Drinking motives
(Bell *et al*. 2023)	Micro-randomized trial	*N* = 350(45.8%) AUDIT score ≥8, interested in drinking less alcohol	Smartphone app, daily report of consumption, notifications, EMA	Self-report (daily)	Engagement
(Fridberg *et al*. 2021)	Within-subject study	*N* = 83(42%) Heavy drinking (≥14/≥7 units p/w male/females), young adults	Smartphone app, 3-h HR-EMA	Self-report	Subjective perceptions
(Glass *et al*. 2017)	Randomized controlled trial	*N* = 349(39.3%) Meeting alcohol dependence criteria (DSM-IV), continuing care	Smartphone app	Self-report (1 month recall)	Treatment seeking
(Giroux *et al*. 2014)	Qualitative	*N* = 26(46.4%) AUD diagnosis, participants from a standalone smartphone-based intervention (LBMI-A)	Smartphone app	Self-report (daily)	User experience
(Glass *et al*. 2022)	Qualitative	*N* = 18(28%)/9(89%) Primary care patients with AUD/primary care clinicians	Potential smartphone app		User experience
(Lang *et al*. 2024)	Mixed method, qualitative	*N* = 11(30%) AUD diagnosis after inpatient treatment, participants from a smartphone-based intervention (Appstinence)	Smartphone app, motivational therapy	Self-report (daily)	User experience
(Wyant *et al*. 2023)	Observational prospective study	*N* = 154(50%) AUD in early recovery	Smartphone app sensors, EMA (4/day)	Self-report (daily)	User experience
(Fridberg *et al*. 2019)	Within-subject study	*N* = 83(42%) Heavy drinking (≥14/≥7 units p/w male/females), young adults	Smartphone app, 3-h HR-EMA	Self-report (day after drinking)	User experience and adherence
(Zetterström *et al*. 2022)	Retrospective study	*N* = 751(?%) AUD diagnosis, data from previous clinical trials, and real-world clinical practice	Smartphone app	Not reported	Validation of other measures
(Hallgren *et al*. 2023)	Single-arm study	*N* = 4121(51.5%) AUD diagnosis, patients from a telehealth program (Ria platform)	Telehealth intervention that uses a breathalyzer	BrAC	Adherence
(Miguel *et al*. 2023)	Within-subject experimental design	*N* = 12(25%) AUDIT score ≥ 8	Contingency management intervention based on a breathalyzer and smartphone app, EMA	BrAC	Adherence
(Liu *et al*. 2023)	Randomized controlled trial	*N* = 51(25.4%) Alcohol dependence diagnosis (DSM-IV), outpatient treatment	Complement intervention	BrAC	Alcohol consumption
(Mitchell *et al*. 2020)	Single-arm study	*N* = 77(48%) AUD diagnosis, patients from a telehealth program (Ria platform)	Telehealth intervention that uses a breathalyzer	BrAC	Alcohol consumption
(You *et al*. 2017)	Prospective cohort study	*N* = 38(?%) Alcohol dependence diagnosis (DSM-IV), outpatient maintenance program	Breathalyzer and eDiary	BrAC	Alcohol consumption
(Lauckner *et al*. 2019)	Prospective observational study	*M* = 17(35%) People living with HIV and meeting alcohol dependence criteria (DSM-IV), HIV clinic patients	Text messages, EMA, daily surveys (2/day), and Breathalyzer	BrAC	User experience
(Hammond *et al*. 2021)	Randomized controlled trial	*N* = 61(39%) AUD diagnosis, community substance use program	Contingency management based on a smartphone app and a breathalyzer.	BrAC	User experience and adherence
(Leeman *et al*. 2022)	Randomized parallel trial	*N* = 99(49%) Young adults reporting heavy drinking (≥4 heavy drinking days, ≥1 eBAC ≥0.10%, ≥10 drinking days, per month)	Breathalyzer, smartphone app, and text messages.	Self-report (daily), BrAC	User experience and adherence
(Oluwoye *et al*. 2019)	Pilot within-subject study	*N* = 6(50%) Risky drinking behavior (≥4/≥3 units in one occasion male/female), nontreatment seeking	Breathalyzer and contingency management	BrAC	User experience and adherence
(Östh *et al*. 2024)	Qualitative	*N* = 21(48%) Alcohol dependence diagnosis (DSM-IV), outpatient treatment	Smartphone apps, one paired with a breathalyzer	Self-report (daily), BrAC	User experience and adherence
(Dermody *et al*. 2018)	Randomized controlled trial	*N* = 58(29%) AUD diagnosis, treatment seeking	SMS message, web-based survey, pill bottle with a chip-embedded cap	Self-report (daily)	Medication adherence
(Hallgren *et al*. 2024)	Randomized controlled trial	*N* = 140(27%) AUD diagnosis, outpatient treatment	Smartphone app, physical activity intervention based on wearable	Self-report (TLFB)	Alcohol consumption
(Walters *et al*. 2021)	Single-arm predictive design	*N* = 78(15.4%) At-risk drinkers (AUDIT score ≥8), receiving health services at a homeless shelter	Ankle-worn sensor and EMA (5/day)	TAC	Alcohol consumption
(Mun *et al*. 2021)	Prospective observational study	*N* = 49(18%) AUDIT score ≥8, receiving health services at a homeless shelter	Ankle-worn sensor and EMA (≥5/day)	Self-report (daily, TLFB), TAC	Alcohol measures
(Leonard *et al*. 2017)	Single-arm study	*N* = 10(100%) Risky drinking behavior (AUDIT score ≥3), nontreatment seeking	EMI and sensor band	Self-report (TLFB)	User experience
(Barnett *et al*. 2017)	Randomized controlled trial	N = 30(46,7%) Heavy drinking (≥14/≥7 units p/w male/females), nontreatment seeking interested in reduced drinking	Contingency management intervention based on wrist-worn sensor, web-based surveys	TAC	Alcohol consumption
(Wang *et al*. 2019)	Qualitative	*N* = ~22 Heavy drinking (≥14/≥7 units p/w male/females), preliminary/qualitative data from other trials	Wrist-worn sensor	TAC (mainly), urine (not specified, BrAC	Monitoring tool experience and validity
Alessi *et al*. 2017)	Qualitative	*N* = 100(40%) AUD diagnostic, outpatient treatment	Ankle-worn sensor	TAC	User experience
(Alessi *et al*. 2019)	Prospective observational study	*N* = 63(41.3%) AUD diagnosis, outpatient treatment	Ankle-worn sensor	TAC	Alcohol consumption
(DiMartini *et al*. 2023)	Pilot randomized controlled trial	*N* = 27(60%) Alcohol-related liver disease, outpatient follow-up after diagnosis or hospitalization	Wrist-worn sensor	TAC	User experience
(Richards *et al*. 2023)	Qualitative	*N* = 20(55%) Heavy drinking (≥2/week drinking), participants of an alcohol contingency management study	Wrist-worn sensor	TAC	User experience

^a^EMA, Ecological Momentary Assessment; BrAC, Breath Alcohol Concentration; TLFB, Timeline Followback; TAC, Transdermal Alcohol Concentration; PEth, phosphatidylethanol; %dCDT, serum carbohydrate–deficient transferrin; GGT, gamma-glutamyl transferase; GGT-CDT = combined index between gamma-glutamyl transferase and carbohydrate-deficient transferrin; EtG, ethyl glucuronide.

Seventeen studies included alcohol consumption as an outcome measure. Of these studies, half were based solely on self-report measures of alcohol consumption, mostly based on daily self-report. Two studies used only a 1-month recall method, such as the Timeline Follow Back (TLFB). The remaining studies predominantly employed remote measurement of alcohol consumption, utilizing both BrAC and TAC as outcome measures, or combined consumption self-report with other alcohol biomarkers. The rest of the studies employed user experience-related outcome measures, such as perceptions of device, application use, and/or treatment adherence as an outcome measure.

### Monitoring through personal smartphones and mobile phones

#### Ecological momentary assessment

Ecological momentary assessment (EMA) was used in 15 studies, making it the most common monitoring method involving smartphones or mobile phones. EMA administers brief self-report prompts throughout the day, enabling data collection in natural settings (Shiffman *et al*. 2008). Compared to long-term recall methods, EMA consumption reports demonstrate stronger correlations with objective alcohol measures (Dulin *et al*. 2017, Mun *et al*. 2021). Studies sent notifications regularly or in specific situations to collect daily data, while some allow self-initiated reporting. Responses were recorded via direct text or voice messages, through hyperlinks to web-based surveys, or directly within smartphone applications.

The use of EMA provided valuable insights into relapse predictors and their interactions during AUD treatment, including motivational patterns, affective states, craving, subjective alcohol sensitivity, and situational triggers (Dvorak *et al*. 2014; Kohen *et al*. 2022; Trela *et al*. 2016; Eddie *et al*. 2021; Emery *et al*. 2022; Maisto *et al*. 2017). For instance, individual states like stress or negative affect elevated the risk of a drinking event and led to increased alcohol consumption (Maisto *et al*. 2017; Dvorak *et al*. 2014; Fridberg *et al*. 2021). However, the likelihood of initiating a drinking episode is shaped by the interaction of individual traits, emotional states, and contextual factors. For example, Walters *et al*. (2021) identified “being inside” and the desire to drink as significant predictors of drinking, while other factors, like “feeling depressed,” contributed to the model but lacked statistical significance by themselves.

EMA was also used to support indirect aspects of AUD treatment, such as medication adherence. While Stoner *et al*. (2015) found that using a smartphone app for daily patient monitoring and sending reminders did not improve naltrexone adherence, Dermody *et al*. (2018) tracked it using text messages and a pill bottle with a chip-embedded cap that automatically logged each opening. Their findings indicate that medication adherence varied daily but decreased over time and was related to assessment compliance, possibly because assessments served as reminders.

One of the major challenges in using EMA is the reporting burden, especially in longer monitoring periods where registers need to be completed regularly (Wyant *et al*. 2023). To address the risk of missing data, some studies have focused on using EMA intensively in specific situations, such as high-resolution EMA (HR-EMA). HR-EMA has proven feasible for intensively monitoring a limited number of drinking events (Fridberg *et al.* 2021; Fridberg *et al*. 2019; Wright *et al*. 2018); still, no study included more than six events in a maximum of 12 weeks.

EMA has been shown to be a feasible and acceptable method for gathering information from real-world settings to monitor alcohol consumption and factors associated with drinking behavior. Reported challenges are related to identifying factors of engagement and response compliance.

#### Passively collected information

The addition of mobile applications in personal smartphones allows the collection of information such as location from Global Positioning System (GPS), or position variation from accelerometer sensors.

Four studies used passively collected GPS information to elicit targeted inquiries in pertinent locations such as bars or pubs where alcohol consumption is likely to occur (Dulin *et al*. 2014, Giroux *et al*. 2014, Gustafson *et al*. 2014, Dulin *et al*. 2017).

Two studies integrated smartphones’ passively collected data with self-reported drinking episodes to predict drinking events. Bae *et al*. (2018) used data collected by a smartphone application installed on heavy-drinking participants’ phones to identify drinking episodes. The researchers compared different machine learning models to determine the most informative sensor features related to a drinking event, which included time variation, movement, device use, and communication patterns. In a later publication with a bigger sample size, Bae *et al*. (2023) reported that their algorithm, using explainable artificial intelligence, was able to predict binge drinking events with 95% accuracy on weekends and 94.3% accuracy on weekdays (Bae *et al*. 2023).

Only one study assessed the acceptability of passive data collection among individuals with AUD (Wyant *et al*. 2023), showing positive perception toward the use of passively collected information. Moreover, participants expressed a greater willingness to engage with predominantly passive data collection methods for a period of 1 year, compared to active methods like self-report.

Passive sensing approaches demonstrated promise for relapse prediction, though evidence remains exploratory. Further research is needed to validate predictors and models across AUD profiles.

### Remote measures from mobile and wearable devices

#### Pocket-sized breathalyzers

Portable breathalyzers are compact devices that allow frequent alcohol monitoring by providing objective measures of alcohol consumption at various times of the day. These devices implement user verification methods through embedded cameras or paired smartphone apps, and this showed to be highly precise and sensitive to small amounts of alcohol (Hämäläinen *et al*. 2018, Leeman *et al*. 2022). Breathalyzer data revealed higher underreporting in long-term recall methods like the TLFB (Kaplan and Koffarnus 2019) and examples of nearly half of self-reports showing abstinence despite measurable alcohol levels in after-care patients (Hämäläinen *et al*. 2020).

The main challenges related to the use of breathalyzer as regular monitoring are the burden on patients, who may need to provide from two to four samples daily (Koffarnus *et al*. 2018, Lauckner *et al*. 2019). While a higher frequency of sampling reduces the risk of missing a drinking event, more frequent sampling and longer monitoring periods increase the risk of missed samples or patient dropout (Hämäläinen *et al*. 2018, Lauckner *et al*. 2019, Hammond *et al*. 2021). Compliance decreased over time, with adherence rates reported from 95.6% in the first month to 51% over a 90-day period (Koffarnus *et al*. 2018, Hallgren *et al*. 2023).

From an alcohol consumption measurement standpoint, missing breathalyzer data represent a key limitation. To address this, trends in completed versus missed samples have been integrated as behavioral indicators (Hämäläinen *et al*. 2020; Zetterström *et al.* 2018). Beyond alcohol intake, in a retrospective study, Hämäläinen *et al.* (2018) observed a rise in missing samples among individuals who eventually dropped out of the program, particularly in the period leading up to their cessation. Zetterström *et al.* (2018) combined missing sample patterns with submission times in prediction models to identify relapse and lapse events, even when a sample was not submitted. A subsequent study based on the same model was able to predict one out of six lapse events using 5 years of retrospective data (Wallden *et al*. 2024).

Breathalyzer measures display higher precision in determining alcohol concentration, but their use still faces challenges related to missing data*.* Although the potential integration of other factors related to the compliance of daily samples has been proposed, further research is necessary to substantiate its efficacy, especially in the context of patients at an elevated risk of non-adherence to therapeutic regimens.

#### Wearables for alcohol concentration measurements

Wearable devices equipped with sensors can estimate alcohol consumption by measuring TAC, typically through wrist and ankle bracelets. Besides providing near-real-time monitoring, the primary advantage of wearable devices lies in their ability to collect data passively, thereby minimizing the risk of missing information due to low adherence to therapy (Goldfine *et al*. 2020).

Similar to breathalyzers, discrepancies between TAC data and long-term recall have been reported. Mun *et al*. (2021) found a weak level of concordance between TLFB (30-day recall) versus TAC and EMA (daily self-reports), with TAC and EMA agreeing on 79% of the alcohol use data. However, EMA faced limitations in accurately identifying drinking days (69.8%) and quantities (59.1%), due to inconsistent reporting. Alessi *et al*. (2019) reported that during a 3-month monitoring period, TAC detected alcohol consumption in over 92.1% of patients, whereas only 46.6% reported consuming alcohol during the same period according to the TLFB.

Limitations of transdermal sensors reported include reduced concurrent validity and user discomfort. TAC measures are less reliable for estimating BAC due to device limitations (e.g. quantification algorithms in development) and individual differences in skin properties (e.g. skin thickness, perspiration) and metabolism (Wang *et al*. 2019). Additionally, TAC peaks are typically delayed and lower than BrAC, reducing sensitivity to detect rapid or low-level alcohol consumption (Alessi *et al*. 2019, Wang *et al*. 2019). While wearables are generally acceptable, some users report discomfort, often from the association of bracelet appearance with justice offenders (Barnett *et al.* 2017; Alessi *et al*. 2017; DiMartini *et al*. 2023). Current research is focused on developing less invasive and more discreet sensor devices, such as subtler wristbands (Wang *et al*. 2019, Gunn *et al*. 2023, Richards *et al*. 2023).

Wearable devices present several advantages, particularly in monitoring alcohol consumption and reducing the compliance burden compared to self-reporting or breathalyzer tests. Challenges include improving user experience, addressing individual variability in TAC measurements, and enhancing the sensitivity to detect varying levels of alcohol.

#### Other devices

In addition to monitoring alcohol consumption, three studies explored technological devices to track parameters related to AUD. Hallgren *et al.* (2024) used a wearable device to monitor physical activity in an intervention aimed at examining how activity levels impact alcohol consumption, finding that both light and regular physical activity were associated with reduced alcohol intake. Alinia *et al*. (2021) employed a wearable device to measure stress, assessing electrodermal activity (EDA) and heart rate variability (HRV). While EDA and HRV were strongly linked to self-reported stress, no significant correlation was found with daily alcohol use or cravings. Finally, Dermody *et al*. (2018) investigated naltrexone adherence using a pill bottle with a chip-embedded cap to track each opening, revealing that adherence declined over time, influenced by daily variations of different factors.

### From remote monitoring to mHealth-based interventions

Most interventions utilized smartphones (*n* = 12), followed by breathalyzers (*n* = 6), with fewer using wearables (*n* = 1) or smart bottles (*n* = 1) (Table 3). Consequently, the strongest evidence of efficacy is found in smartphone-based interventions, particularly the addiction comprehensive health enhancement support system (A-CHESS) system, which has been evaluated in three RCTs, followed by breathalyzer-based interventions.

**Table 3:** Summary of interventions, devices, and related studies. The table outlines interventions involving different mHealth devices used in AUD studies

**Intervention**	**Device**	**Description**	**Related studies**
Contingency management	Wearable (SCRAM)	Standalone contingency management intervention, escalating cash reinforcement for alcohol not reported or detected based on TAC measures	Barnett *et al*. 2017 ^a^
Contingency management	Breathalyzer (Soberlink)	Standalone contingency management intervention, 3 samples per day, ID verification through breathalyzer- embedded camera, escalating cash reinforcement based on submitted sample and no detected alcohol based on BrAC measures. Patients are required to provide a monetary deposit in Koffarnus *et al*. (2021).	Koffarnus *et al*. 2018 (parent study)^b^; Koffarnus *et al*. 2021^b^; Kaplan and Koffarnus 2019^b^
ARMS	Smartphone (ARMS app), breathalyzer (BACtrack)	Standalone contingency management intervention, 3 samples per day, ID verification through photo uploaded in the app, automated reinforcement management system based on BrAC measures; it includes the use from EMA and GPS.	Miguel *et al*. 2023 ^c^
Contingency management	Smartphone (BACtrack View app), breathalyzer (BACtrack)	Standalone contingency management intervention, 2–8 samples per day, ID verification through photo uploaded in the app; submitted and/or negative samples are monetary-reinforced based on BrAC measures.	Oluwoye *et al.* 2019 ^c^
DynamiCare	Smartphone (DynamiCare Health), breathalyzer (BACtrack)	Contingency management intervention as a complement to outpatient care, 1–3 samples per day, ID verification through video uploaded in the app, reinforcement based on submitted sample and no detected alcohol based on BrAC measures.	Hammond *et al*. 2021 ^a^
A-CHESS	Smartphone (A-CHESS app)	Complement to outpatient care focused on relapse prevention; the app includes a panic button, daily reports, relaxation exercises, games, informative resources, and GPS-driven information. Based on inactivity or provided answers, a relapse risk is calculated. It included a clinician’s portal.	McKay *et al*. 2022 ^a^; Glass *et al*. 2017^a^; Gustafson *et al.* 2014^a^
AGATE-Rx	Smartphone (AGATE-Rx app), pill bottle with a chip-embedded cap (MEMS)	Standalone intervention focused on monitoring medication adherence; daily reminders sent via SMS.	Dermody *et al*. 2018 ^a^; Stoner *et al*. 2015^a^
Appstinence	Smartphone (Appstinence app)	Complement to aftercare intervention based on telephone coaching, the app included a chat to communicate with coaching and modules with resources.	Lang *et al*. 2024
HealthCall	Smartphone (HealthCall app)	Complement to outpatient care based on motivational interventions or a clinical guide, the app included daily reports and provided personalized feedback.	Hasin *et al*. 2022 ^b^
Journaling app	Smartphone	Complement to outpatient care, the app included daily reporting; based on the reports, an average alcohol intake was calculated. It included a clinician’s portal.	Yamashiki *et al*. 2023 ^c^
LBMI-A	Smartphone (Buddy Tool app)	Standalone, self-paced intervention, the app includes psychoeducation modules, a drink monitor tool, fast access to support contact, GPS-driven information, and a craving tool to report craving to drink from which a coping option is displayed.	Dulin *et al*. 2017 ^b^; Dulin *et al*. 2014^c^; Giroux *et al*. 2014
Ria Platform	Smartphone (Ria platform), breathalyzer (BACtrack)	Standalone remote intervention based on telehealth, 2 samples per day, the app sends reminders to provide samples and reports. It includes a clinician’s portal.	Mitchell *et al*. 2020 ^c^: Hallgren *et al*. 2023^c^
Previct Alcohol	Smartphone (Previct alcohol), breathalyzer (Kontigo care)	eHealth support system, complement to outpatient care. The app included questionnaires, reported craving, therapeutic tasks, and a help button. ID verification through a breathalyzer-embedded camera. Based on the collected information, a relapse risk warning was sent to the patient and caregiver, through the app and a clinician’s portal.	Wallden *et al*. 2024; Zetterström *et al*. 2022; Zetterström *et al*. 2021; Hämäläinen *et al*. 2020^a^; Zetterström *et al*. 2018; Hämäläinen *et al*. 2018^c^
SoberDiary	Smartphone (SoberDiary), breathalyzer (SoberLink)	Complement to outpatient care, based in journaling. App includes daily reports and modules with resources. Patients receive points for adherence (i.e. number of breath samples, access to the app and use of the modules).	Liu *et al*. 2023 ^a^; You *et al*. 2017^c^
DrinkLess	Smartphone (Drink less app)	Standalone intervention to reduce alcohol consumption. The app includes notifications to complete daily reports and provides modules with psychoeducational information for behavioral change.	Bell *et al*. 2023 ^c^
Mind the Moment	Smartphone (MtM app), wearable (Empatica E4)	Standalone intervention-based motivational interviewing and cognitive behavioral therapy. Measures of EDA were used to provide a notification of stress or arousal status through vibration. The app included EDA reports, questionnaires, and tailored coping strategies.	Leonard *et al*. 2017 ^c^
Glasklart	Smartphone (app)	Complement to outpatient care. The app includes a daily self-report of consumption, mood, location and social context, and a progress overview. If consumption is reported, a notification is prompted to report additional units. It includes a healthcare portal.	Östh *et al*. 2024
iBAC	Smartphone (iBAC), breathalyzer	Complement to outpatient care. The app provided notifications to provide a breath sample 3 times a day, to report their subjective mood and an overview of their results. ID verification through photo uploaded in the app. Iit includes a clinician’s portal.	Östh *et al*. 2024
Smart-T alcohol	Smartphone (app)	Standalone JITAI, based on daily self-report. The app provides motivational messages on demand, assesses momentary risk factors for imminent drinking using EMA, and provides tailored messages based on individual and contextual factors.	Walters *et al*. 2022 ^c^
SMS intervention	Smartphone, mobile phone	Standalone SMS intervention, delivering motivational and coping messages daily. Uses brief self-report prompts to assess mood, craving, and relapse risk, with tailored responses based on input. Designed to support abstinence and stress reduction in liver transplant candidates.	DeMartini *et al*. 2018
EMA-based intervention	Smartphone	Standalone JITAI, based on event-level self-report. Participants completed EMAs during drinking events, triggering tailored SMS feedback based on mood, location, intentions, and cumulative consumption. Messages were motivational and context-sensitive, delivered hourly throughout the night to reduce harm and support safer drinking behavior.	Wright *et al*. 2018 ^a^

^a^RCT, randomized controlled trial;

^b^RPT, randomized parallel trial;

^c^CT, clinical trial.

#### Mobile phone– and smartphone-based interventions

Smartphone-based intervention components included text message support, personalized notifications, and psychoeducational resources. Text message content was customized in two interventions (A-CHESS and the location-based monitoring and intervention of alcohol use disorders (LBMI-A)) based on intake information, prior EMA responses, passively collected data (e.g. GPS location), or a combination of these elements. In contrast, a study by Yamashiki *et al*. (2023) focused on a journaling-based intervention with reminders for daily diary entries but without additional notifications or messages.

Most mHealth interventions reduced alcohol consumption in single-arm studies (Dulin *et al*. 2014, Yamashiki *et al*. 2023). As complement to TAU conditions, they produced better outcomes than TAU alone in a pilot RCT (DeMartini *et al*. 2018), a three-armed randomized trial (Hasin *et al*. 2022), and two RCTs (Gustafson *et al*. 2014: McKay *et al*. 2022). Wright *et al*. (2018) found no difference in alcohol use between an ecological momentary intervention based on HR-EMA and control. McKay *et al*. (2022) found no significant difference between the telephone-based intervention and A-CHESS, suggesting that the mHealth approach achieved similar outcomes while requiring minimal clinical supervision. Other results report an increased use of coping strategies (Dulin *et al*. 2017) and enhance the engagement with outpatient services (Glass *et al*. 2017).

#### Interventions paired with devices

Four contingency management (CM) studies showed how pocket-sized breathalyzers and transdermal sensors addressed logistical challenges in rewarding alcohol abstinence. In CM, tangible rewards (e.g. monetary vouchers) are provided to reinforce abstinence (Petry *et al*. 2000). Most CM interventions used breathalyzers with incentives tied to performed sample and/or negative test results, effectively reducing alcohol consumption (Koffarnus *et al*. 2018, Oluwoye *et al*. 2019, Koffarnus *et al*. 2021, Miguel *et al*. 2023). For example, Koffarnus *et al*. (2021) reported 86% abstinence in the contingent group compared to 44% in the noncontingent group. CM also improved retention and compliance in breathalyzer use (Oluwoye *et al.* 2019; Hammond *et al*. 2021). One RCT with a wearable device showed initial gains in alcohol-free days for the CM group, but the effect did not persist at follow-up (Barnett *et al*. 2017).

One standalone eHealth intervention (Ria Platform) showed reduced alcohol consumption at the end of treatment in a clinical setting (Mitchell *et al*. 2020). Two support systems for outpatient care, SoberDiary and Previct Alcohol, were evaluated in RCTs, which found that breathalyzers alone did not improve outcomes compared to TAU conditions (Hämäläinen *et al*. 2020, Liu *et al*. 2023). Better clinical outcomes were linked to higher compliance, with patients who missed fewer samples showing better clinical results (You *et al*. 2017, Hallgren *et al*. 2023).

#### mHealth as an information source for clinicians

Three interventions incorporated risk indicators for care providers, leveraging predictive models to synthesize multiple data sources on an ongoing basis. For example, the Automated Reinforcement Management System (ARMS) includes a “relapse risk score” derived from EMA, GPS, and BrAC results to alert practitioners to an increased risk of relapse (Miguel *et al*. 2023). Similar to ARMS, the Previct Alcohol intervention utilizes an algorithm that predicts relapse risk within the next 3 days based on BrAC and questionnaire results, therapy compliance, craving levels, and other unspecified factors (Wallden *et al*. 2024). Among these studies, only Previct Alcohol reported clinical implementation outcomes in a retrospective study, which showed that sending a “risk warning” to clinicians reduced relapse quantity and duration by 9%–18%. The impact on duration may be attributed to the advantage of early risk notification, which helps prevent longer and more pervasive relapse events.

#### Toward Just-In-Time Adaptive Interventions

Identifying pertinent momentary risk factors is crucial for developing intervention strategies that can be implemented precisely when they are most needed, such as through Just-In-Time Adaptive Interventions (JITAIs). The objective of the JITAI approach is to provide the right type of support at the optimal time, based on continuous adaptation to the patient’s needs as informed by a constant stream of data (Marsch 2020). Unlike static, linear intervention processes, JITAIs follow a dynamic and iterative approach, tailoring support to the individual’s current state (Nahum-Shani *et al*. 2023). This approach ensures that interventions are delivered at the most opportune moment, thereby avoiding unnecessary or less effective interventions.

Our search identified one JITAI intervention based on EMA aimed at addressing alcohol misuse in adults experiencing homelessness (Walters *et al*. 2022). Results showed that the intervention was well accepted and that the 4-week program contributed to a reduction in alcohol consumption. Participants also had a positive perception of message frequency and frequently utilized the app’s available resources. However, these results are considered preliminary due to the short duration of the intervention and the absence of a comparison group.

Other studies included in this review have investigated components that could potentially be integrated into JITAI interventions. For instance, the development of prediction models that identify the risk of drinking within a day (Bae *et al*. 2018, Walters *et al*. 2021, Bae *et al*. 2023) or the use of location data to pinpoint high-risk contexts (Dulin *et al*. 2017) can help identify critical windows for deploying momentary interventions. Additionally, Bell *et al*. (2023) showed how notifications benefit user engagement; however, further research into dynamic components is required.

### User experience

Fifteen studies had user experience on remote monitoring tools as the main outcome measures. Three studies focused on potential developments and desired characteristics for these tools (Gordon *et al*. 2017, Glass *et al*. 2022, Richards *et al*. 2023), while the majority collected feedback on tools already implemented. Among these, the longest reported period of use was 16 weeks with wearables (DiMartini *et al*. 2023) and most remote monitoring tools were reported as acceptable and well tolerated by participants (Giroux *et al*. 2014, Alessi *et al*. 2017, Leonard *et al*. 2017, DeMartini *et al*. 2018, Lauckner *et al*. 2019, Hammond *et al*. 2021, DiMartini *et al*. 2023, Wyant *et al*. 2023, Lang *et al*. 2024).

Studies highlight several key barriers to using smartphone apps or devices for monitoring alcohol consumption. These barriers include technical issues (Oluwoye *et al.* 2019; Lauckner *et al*. 2019; Östh *et al*. 2024) and concerns related to the stigma associated with the appearance of monitoring tools (Alessi *et al*. 2017; Lauckner *et al.* 2019; Richards *et al*. 2023; Östh *et al*. 2024; Leeman *et al*. 2022). Stigma-related concerns emphasize the need for discrete support tools, both in terms of appearance and names. For apps used as a complement to treatment, patient engagement is referred to depends on the level of clinician involvement in monitoring their progress (Glass *et al*. 2022). Clinicians themselves showed positive perception of monitoring tools; however, the need of operational adaptations for these tools to be effectively integrated into care is reported (Gordon *et al*. 2017, Glass *et al*. 2022).

Two studies reported that a smartphone app to monitor alcohol consumption increased participants’ awareness of their alcohol problem and improved the accuracy of their self-reported consumption (Giroux *et al*. 2014, Östh *et al*. 2024). Additionally, the use of breathalyzers was reported to boost treatment motivation and facilitate the treatment process, similar to the effects observed with transdermal monitors (Alessi *et al*. 2019, Lauckner *et al*. 2019, Buono *et al*. 2023). Participants also reported an increase in their awareness of stressors and drinking behavior when physical feedback was provided by the wearable (Leonard *et al*. 2017).

## Discussion

This review explores the current state of mHealth technologies for the remote monitoring of AUD and the development of new interventions based on constant data streams. As an addition to previous reviews on this topic, we provide a comprehensive synthesis of the available evidence, incorporating diverse methodological approaches to offer an updated overview of the field of AUD.

Smartphones, breathalyzers, and wearables with transdermal sensors were the most frequently used devices for remote monitoring with an uneven level of evidence. Smartphone-based interventions were the most extensively studied, with many utilizing EMA to capture beliefs, motivation, craving, and affective states in real time. EMA has proven valuable for identifying relapse predictors and improving the accuracy of self-reported alcohol use by minimizing recall bias. However, missing data remain a challenge, similar to breathalyzer-based systems. Breathalyzers were frequently tested in clinical trials and remote interventions such as contingency management and continuing care, showing evidence of efficacy across multiple approaches. Transdermal sensors offer the advantage of passive and continuous data collection, potentially addressing the issue of missing data, yet the current evidence remains limited, focusing primarily on feasibility and user experience. Recent developments involving passive smartphone sensing and predictive modeling show potential for near real-time relapse prediction and tailored interventions, besides reducing the risk of missing data, but remain in the exploratory phase. Overall, smartphone-based interventions show the strongest and most consistent support for efficacy concentrated in one specific intervention (A-CHESS), while evidence from breathalyzer studies suggests broader applicability across intervention models. Further research is needed to strengthen the evidence base for newer and less-tested technologies such as wearables and passive data systems.

Our review highlights the feasibility of long-term monitoring using technology, particularly through smartphone apps for regular check-ups and breathalyzer-based systems. These approaches demonstrate that extended monitoring periods (>6 months) are feasible. Nonetheless, challenges related to missing data increase as the duration of monitoring extends. While these challenges were addressed by adapting reporting frequencies based on contextual information and utilizing predictive models to assess compliance variations, only one study directly assessed engagement factors.

The implementation of objective measures of alcohol using breathalyzers and wearables also showed validity advantages compared with traditional monitoring tools, like the TLFB. These findings underscore the value of objective measures in assessing treatment outcomes, as results may vary depending on the measurement method.

Beyond the use of individual devices, there is a limited number of studies that combine multiple monitoring strategies. Integrating diverse data sources could offer a more comprehensive understanding of alcohol consumption dynamics while managing the burden of information collection. EMA provides valuable insights into internal and subjective states, passive smartphone data offer contextual and activity-related information, and biosensors deliver objective measures of drinking status. mHealth monitoring systems should be tailored to individual patient profiles, and therapeutic stages may enhance both efficacy and engagement.

Most mHealth tools were intended as complements to outpatient interventions, but only a few studies reported how the collected data were integrated into clinical practice. Some included clinician dashboards summarizing patient status, apps enabling communication, or risk indicators. However, no study detailed how clinicians utilized this information. Clear guidelines for implementing real-world data could help tailor therapy sessions to individual needs, targeting specific challenges and high-risk situations. Notably, one study found that an mHealth intervention requiring minimal clinical supervision produced outcomes comparable to a phone-based intervention. Considering clinical workload alongside efficacy may highlight the potential of mHealth tools to improve accessibility in addiction care.

Qualitative studies highlighted insights from patients and clinicians when using mHealth resources. The findings indicate an overall positive perception of these tools, though long-term engagement remains a significant challenge. Results also show the importance of stigma in the user design of monitoring systems, especially when they are deployed in public spaces, and the therapeutic effect of monitoring by itself to keep motivation in treatment. Further research should explore long-term monitoring perceptions, challenges, and potential adaptations to minimize missed samples.

While not the primary focus of this review, the issue of data security emerged as a significant concern. Privacy and data protection must be addressed explicitly, as concerns about surveillance can also impact patient participation. Future research should include robust security measures to protect personal information (Nurgalieva and Doherty 2023).

This review was subject to several limitations. Firstly, a standardized tool to assess risk of bias was not included, which may limit the quality assessment of the included studies. This decision was made because the primary aim of this integrative review was to provide an overview of the state of the art in an emerging field, requiring a broader scope than a traditional systematic review. Secondly, our focus on individuals with AUD-related disorders based on explicit diagnostic criteria and tools such as the Alcohol Use Disorders Identification Test (AUDIT) excluded relevant studies addressing hazardous alcohol consumption in social settings. This focus was driven by the need to address the specific challenges faced by this population, given current treatment limitations. Lastly, this review did not examine different therapeutic approaches within monitoring processes and intervention outcomes. The studies included employed varying roles for mHealth tools, from fully digital interventions to blended approaches with traditional care. Additionally, variability in clinical involvement was not systematically coded due to heterogeneity in study aims and insufficient details.

## Conclusion

mHealth-supported monitoring systems show strong potential to improve AUD treatment, yet evidence remains uneven. Smartphones are the most studied, with breathalyzers showing promise for clinical integration. Wearable sensors have limited clinical use, while passive data collection is a promising but emerging approach. This integrative review highlights both progress and persistent gaps, emphasizing the need for more robust evidence to support the selection and integration of these tools into routine care.

## Data Availability

This article is an integrative review based on previously published studies. Coded summary data used to generate the tables are available from the corresponding author upon reasonable request.
